# EMD-Based Method for Supervised Classification of Parkinson’s Disease Patients Using Balance Control Data

**DOI:** 10.3390/bioengineering9070283

**Published:** 2022-06-28

**Authors:** Khaled Safi, Wael Hosny Fouad Aly, Mouhammad AlAkkoumi, Hassan Kanj, Mouna Ghedira, Emilie Hutin

**Affiliations:** 1 Computer Science Department, Strasbourg University, 67081 Strasbourg, France; 2 College of Engineering and Technology, American University of the Middle East, Egaila 54200, Kuwait; wael.aly@aum.edu.kw (W.H.F.A.); mouhammad.a@aum.edu.kw (M.A.); hassan.kanj@aum.edu.kw (H.K.); 3 Laboratory ARM, EA BIOTN, UPEC, CHU Henri Mondor, 94000 Cŕeteil, France; mouna.ghedira@aphp.fr (M.G.); emilie.hutin@aphp.fr (E.H.)

**Keywords:** feature extraction, feature selection, machine learning, Parkinson’s disease, postural stability, stabilometric data

## Abstract

There has recently been increasing interest in postural stability aimed at gaining a better understanding of the human postural system. This system controls human balance in quiet standing and during locomotion. Parkinson’s disease (PD) is the most common degenerative movement disorder that affects human stability and causes falls and injuries. This paper proposes a novel methodology to differentiate between healthy individuals and those with PD through the empirical mode decomposition (EMD) method. EMD enables the breaking down of a complex signal into several elementary signals called intrinsic mode functions (IMFs). Three temporal parameters and three spectral parameters are extracted from each stabilometric signal as well as from its IMFs. Next, the best five features are selected using the feature selection method. The classification task is carried out using four known machine-learning methods, KNN, decision tree, Random Forest and SVM classifiers, over 10-fold cross validation. The used dataset consists of 28 healthy subjects (14 young adults and 14 old adults) and 32 PD patients (12 young adults and 20 old adults). The SVM method has a performance of 92% and the Dempster–Sahfer formalism method has an accuracy of 96.51%.

## 1. Introduction

The key function of the human postural system is to stabilize the human body in any static or moving form. This is accomplished by considering external perturbations for the static posture that is also called quiet standing as well as during locomotion. The human postural system uses the interactions between the central nervous system, the musculoskeletal system and three sensory systems, the vestibular, visual and proprioception systems, to maintain the body in its upright position [1,2,3,4,5].

Parkinson’s disease (PD) is one of the most common movement disorder diseases that cause damage in the nervous system. As a result, human postural stability is affected and the human is more susceptible to suffering physical injuries. The main cause of this disease is degradation of the motor control and malfunctioning of the rhythm generation in the basal ganglia. This affects the postural stability during quiet standing and locomotion [6,7].

PD has been the subject of many research studies focused on quiet standing and dynamic postures [8,9,10,11,12]. Data-mining techniques can be used for feature extraction from collected data to provide the classification [13] of PD and non-PD subjects [14,15].

Center-of-pressure (COP) displacements are used to analyse and evaluate the postural stability of the human body in quiet standing. COP displacements are usually recorded in two directions, in the right/left (medial–lateral) direction and in the forward/backward (anterior–posterior) direction of the human body.

Analyzed center-of-pressure (COP) output measures usually lack sensitivity. Thus, the standard spatiotemporal analysis of the COP may provide only descriptive information without any direct insight into underlying control deficits.

In [16,17], Stodolka Tanaka et al. proposed a new methodology to assess postural stability through the center-of-pressure (COP) trajectories during quiet standing. New sensitive parameters were extracted and then utilized to investigate changes in postural stability with respect to visual input. The experimental data consists of stabilometric signals of eleven healthy subjects (20–27 years). These signals were recorded under eyes-open and eyes-closed conditions using a force platform during quiet standing. The proposed approach was applied separately for medial–lateral and anterior–posterior stabilometric signals.

In [17], the stabilometric signals were modeled for each subject and for each condition as an auto-regressive (AR) model. This is achieved for each direction (medial–lateral (ML) and anterior–posterior (AP)) separately, and the order of the AR models was practically fixed at M = 20. The new measures (the percentage contributions and geometrical moment of AR coefficients) were obtained from the estimation of the AR model parameters. They showed statistically significant differences between open-eyes and closed-eyes conditions. The quiet standing under eyes-open conditions showed higher correlation between present and past COP displacements compared to the eyes-closed conditions. In contrast, no significant differences between vision conditions were found for conventional classical parameters (the total length of the COP path, mean velocity). The results showed that the AR parameters are useful for assessing postural stability during static posture for visual conditions.

In [14], Palmerini et al. used accelerometer-based data recorded from control and PD subjects to analyze posture in a quiet stance. First, 175 measures were computed from time and frequency domains, and feature selections with classification techniques were then used to select the best parameters that discriminate between control and PD subjects. Two parameters were selected to clearly separate the control subjects from the PD subjects. Note that the feature extraction, feature selection and training phases generally require additional computational time, which can be annoying for real-time analysis.

In an attempt to diverge from the standard COP characteristics approach, Blaszczyk assessed human postural stability by using force-plate posturography [18]. The work focused on a dataset consisting of 168 subjects who were grouped into three categories: young adults, older adults and patients with PD. The subjects were requested to stand still and to have their eyes open and then to stand still with their eyes closed. To better understand postural stability, the authors introduced three new output measurements: the sway ratio (SR), the sway directional index (DI) and the sway vector (SV). The inputs to the system were: age, pathology and visual conditions. These variables highly affected the measured outputs of the method. They resulted in distinctive differences between eyes-open and eyes-closed groups, young adults and old adults groups, young adults and PD groups, and between old adults and PD groups. As a conclusion to this work, the use of sway vector is recommended as a suitable variable in assessing postural control in quiet standing.

### Empirical Mode Decomposition

In 1998, Norden Huang, a NASA engineer, proposed a nonlinear method called Empirical Mode Decomposition (EMD) to analyze nonlinear and non-stationary signals [19,20,21]. This method is comparable with Fourier and wavelet transforms where a signal is composed of several elementary signals. Different than other methods, EMD breaks up any given signal into a finite number (N) of oscillating components extracted directly without any a priori condition. The resulting components are the intrinsic mode functions (IMFs). These functions are non-stationary wave-forms. The range of frequencies from highest to lowest present in the signal is represented by the IMFs. The IMFs are oscillating functions that have zero mean.

Any signal can be written as:

(1) $$Signal=\sum_{k=1}^{N}IMF_{k}+r_{K}$$

where $IMF_{k}$ is the *k*th IMF and $r_{K}$ is the residual signal.

An IMF (intrinsic mode function) is an amplitude-modulated and frequency-modulated signal that is represented with the following characteristics:

1. The number of local maxima and the number of local minima differ at most by one or are equal otherwise.
2. The mean of the lower and upper envelopes is approximately null everywhere.

EMD is an iterative approach where the first component (IMF) is obtained from the original signal and the estimation of the second IMF is obtained from the residual signal, and so on. The significance of the EMD method in our methodology is based on the fact that this method decomposes the stabilometric signal into elementary signals based on frequency bands. This enables us to analyze, separately, each signal (IMF) that holds a specific frequency band. This strategy gives information in depth about the characteristics of a the stabilometric signal to reach the best classification results.

## 2. Supervised Machine-Learning Approaches

Machine learning (ML) is a sub-category of the artificial-intelligence (AI) technique used to increase the system knowledge [22]. The ML technique provides computers with the ability to learn autonomously. It is mainly categorized into three categories: (1) supervised, (2) unsupervised and (3) semi-supervised learning approaches [23]. Supervised algorithms (SAs) takes the needed input and outputs from humans. During the training process, an SA provides feedback about the prediction accuracy. SA is widely used in data classification process for different applications such as early detection and prediction of diabetes [24,25,26,27], prediction of Alzheimer’s Disease [28,29,30,31], detection of Acute Respiratory Distress Syndrome [32,33,34] and EEG Signal Processing [35,36,37,38].

This section introduces and discusses briefly the main supervised learning approaches.

KNN

One of the simplest and high performing methods used in supervised classification is k-nearest neighbors (KNN) [39]. It is one of the applied non-parametric approaches that are used in different systems such as weather prediction [40], facial expression classification [41], eye movement detection [42] and prediction of hospital readmission for diabetic patients [43]. For this method, the classification of a new individual occurs by:

(1) Computing the distance between this individual and all other individuals in the dataset used for training. This distance function is the similarity measure for the new case.
(2) Choosing the most common class among k-nearest neighbors for the new case in order to be able to classify it.

CART

The classification and regression tree (CART) method is referred to as the decision tree algorithm commonly used in machine learning [44]. The high interest in using this method is due to the simplicity, efficiency and easy interpretation of the algorithm. The algorithm identifies the non-linear relationships between the input and outputs of a given system. The decision tree is composed of nodes and branches classifying the variables in recursive partitions. The leaf node does not project any branching. This method was successfully implemented in many fields [45,46].

RF

The Random Forests are a family of methods which consist of the construction, as its name suggests, of a set (or forest) of decision trees. In [47], Breiman combines the bagging method which is an abbreviation of “bootstrap aggregating” and the random selection of the partitioning variable of each node to give a new method called random forests [48,49,50].

Combining these processes improves the classification performance of a single tree classifier [51,52]. The assignment of a new observation vector to a class is based on a majority vote of the different decisions provided by each tree constituting the forest.

SVM

Support Vector Machine (SVM) is another widely recognized and widely used method for supervised learning due to its high accuracy [53,54]. It is usually ranked among the best classifiers giving the best results for resolving binary discrimination and classification problems [55,56,57]. The objective of SVM is to determine a hyperplane in an N-dimensional space to classify the given data points. For example, if the data is linearly separable, then we find the hyperplane (separator) $f\left(x\right)=w^{T}x+b$ that differentiates the positive observations ($y_{i}=+1$) from the negative observations ($y_{i}=-1$). It also maximizes as much as possible the distance between the support vectors and the hyperplane. The margin SVM should be equal to twice the distance between the hyperplane and the support vectors.

## 3. Methodology

In this section, we introduce the data acquisition process; then, we explain the proposed methodology that is used for the classification of the healthy subjects and those with PD.

### 3.1. Data Acquisition

The Mondor Hospital in Creteil, France, was the facility where the data were acquired and the experiments were conducted. The resulting dataset is extracted from 28 healthy subjects (14 young adults and 14 old adults) and 32 PD patients (12 young adults and 20 old adults). The dataset included stabilometric signals from those 60 specimens. Details about the PD population are represented in Table 1.

Healthy and PD individuals were requested to perform quiet standing during the recording trials of the stabilometric signals in the AP and ML directions for 60 s. ML trajectories are the center-of-pressure movements in the right/left directions of the human body. AP trajectories are the center-of-pressure movements in the forward/backward directions.

A six-components force plate (60 × 40 cm, strain-gauge-based device from Bertec Corporation, Columbus, OH, USA) with sampling rate of 1000 Hz is used to do the measurements.

### 3.2. Proposed Classification Process

**Figure 1:**
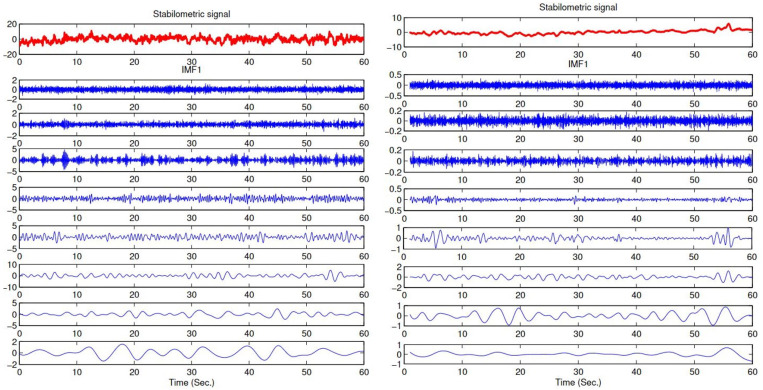
AP stabilometric signal and its first eight IMFs for PD (**left**) and healthy (**right**) subjects.

The method is applied by extracting the EMD-based temporal and spectral features from the stabilometric signals. MATLAB programming language is used to implement all stages of this methodology. Four main stages are needed in this process:

1. Breaking down the stabilometric signal using EMD, and obtaining a set of IMFs. The first eight IMFs are used in processing and in feature extraction, see Figure 1.
2. Feature extraction: extract three time-domain features, standard deviation ($\sigma_{t}$), skewness ($\beta_{t}$) and kurtosis ($Kurt_{t}$), and then three frequency-domain features, spectral centroid ($C_{s}$), spectral skewness ($\beta_{s}$) and spectral kurtosis ($Kurt_{s}$). These features are extracted from the stabilometric signals and from their IMFs to compare the classification results.
3. Characteristics selection: selecting the first five relevant characteristics that represent the postural sway of healthy subjects and subjects with PD using the random forest algorithm.
4. Machine-learning applications: using the four machine-learning approaches described before: KNN, CART, RF and Support Vector Machine (SVM) for classifying the healthy subjects and subjects with PD using 10-fold cross validation.

In summary, The importance of the methodology is shown in having the EMD method decompose the Stabilometric signal based on frequency bands. This helps to analyze, separately, each signal that holds a specific frequency band (IMF). We use temporal and frequency features to extract both spectral and temporary behaviors for each frequency band (IMF) from the original signal. This gives information in depth about the characteristics of a stabilometric signal to reach the best classification results.

**Figure 2:**
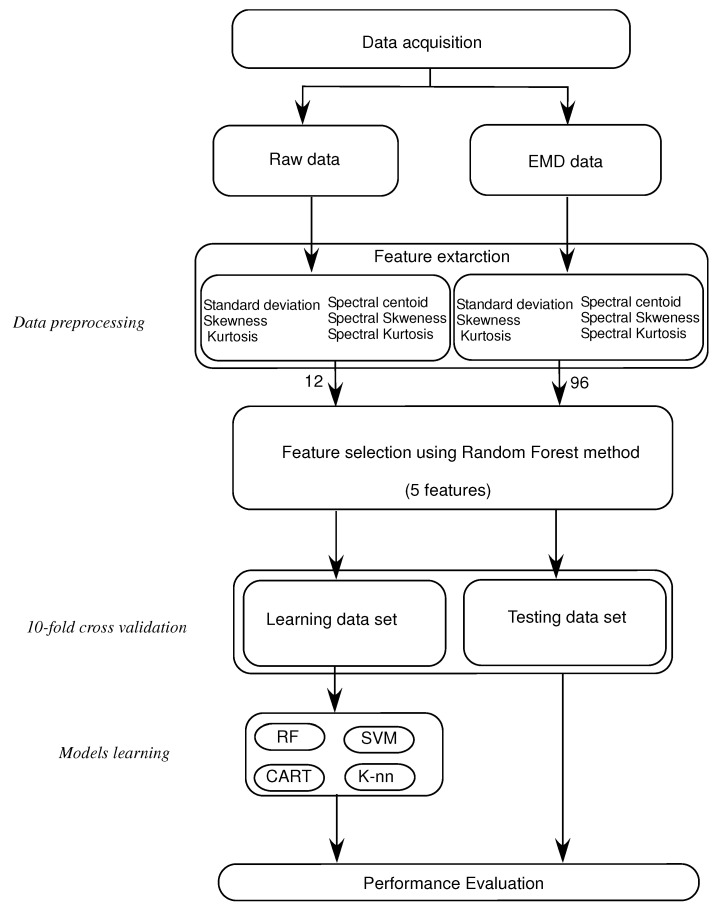
Healthy and PD patient classification process.

Figure 2 shows the process of the proposed approach for classifying the healthy subjects and subjects with PD.

### 3.3. Features Extraction

The three temporal features studied in this research are: the standard deviation $\sigma_{t}$ of the signal as stated in Equation (2). Equation (3) shows how to calculate the skewness $\beta_{t}$, which is the variable that evaluates the asymmetry of the probability distribution of the data.

Kurtosis $Kurt_{t}$ is another measurement that finds the tailedness of the probability distribution, as illustrated in Equation (4).

(2) $$\sigma_{t}=\sqrt{\frac{1}{N}\sum_{i=1}^{N}{\left(x\left(i\right)-\mu \right)}^{2}}$$

where *N* is the number of samples in a given signal *x*, and μ is the mean value.

(3) $$\beta_{t}=\frac{1}{N}\sum_{i=1}^{N}{\left(\frac{x(i)-\mu }{\sigma }\right)}^{3}$$

(4) $$Kurt_{t}=\frac{1}{N}\sum_{i=1}^{N}{\left(\frac{x(i)-\mu }{\sigma }\right)}^{4}$$

Next, three spectral features are extracted form the stabilometric signal itself and from its IMFs.

The spectral energy distribution of the data is simply characterised by the features extracted from the signal. Equation (5) shows the spectral centroid $C_{s}$, which is the center of mass of the spectrum that is commonly used in connection with the brightness of sound and for analysis of the musical timbre.

To calculate the tailedness and the asymmetry of the spectral energy distribution, the spectral kurtosis $Kurt_{s}$ and the spectral skewness $\beta_{s}$ are used as shown in Equations (6) and (7).

(5) $$C_{s}=\frac{\sum_{w}wP\left(w\right)}{\sum_{w}P\left(w\right)}$$

where P(w) is the amplitude of the w frequency bin of the spectrum.

(6) $$\beta_{s}=\frac{\sum_{w}{\left(\frac{w-C_{s}}{\sigma_{s}}\right)}^{3}P\left(w\right)}{\sum_{w}P\left(w\right)}$$

where $Sigma_{s}$ is the mean square root of the spectral variation.

(7) $$Kurt_{s}=\frac{\sum_{w}{\left(\frac{w-C_{s}}{\sigma_{s}}\right)}^{4}P\left(w\right)}{\sum_{w}P\left(w\right)}$$

### 3.4. Performance Evaluation

The performance evaluation of the proposed method lies under the ability of classifying healthy subjects and subjects with PD. To achieve this evaluation, four key elements should be measured. These elements depend on the number of true positives and true negatives as well as the number of false positives and false negatives. The elements are:

1—Accuracy, which is calculated using Equation (8).

(8) $$Accuracy=\frac{T_{p}+T_{n}}{T_{p}+T_{n}+F_{p}+F_{n}},$$

2—Recall, which is calculated using Equation (9).

(9) $$recall=\frac{T_{p}}{T_{p}+F_{p}},$$

3—Precision criteria, which is calculated using Equation (10).

(10) $$precision=\frac{T_{p}}{T_{p}+F_{n}},$$

where:

- $T_{p}$ represents the number of true positive examples;
- $T_{n}$ represents the number of true negative examples;
- $F_{p}$ represents the number of false positive examples;
- $F_{n}$ represents the number of false negative examples.

4—F-measure, which is calculated using Equation (11).

(11) $$F_{\beta }-measure=\frac{(1+\beta^{2}).recall.precision}{\beta^{2}recall+precision},$$

where $F_{\beta }$ is a special measure to focus more on the precision and recall for a given single score. To be able to give equal importance to the precision and recall, β is given the value 1.

A supervised learning strategy is used with the stabilometric data to classify healthy subjects and subjects with PD. As such, the data labels were utilized in both the training stage and the testing stage of the models. In our approach, a 10-fold cross-validation method was used to generate the training dataset and the testing dataset.

## 4. Experimental Results

### 4.1. Results and Discussions

As part of the proposed method, stabilometric signals are first decomposed to IMFs signals using the EMD method. Next, the feature extraction step is performed on raw data and EMD data (IMFs) to compare the obtained results as descriptive results and results for our proposed study, respectively. The features are categorized into two groups: the first is a group of three time-domain features, standard deviation ($\sigma_{t}$), skewness ($\beta_{t}$) and kurtosis ($Kurt_{t}$), and the second groups consists of three frequency-domain features, spectral centroid ($C_{s}$), spectral skewness ($\beta_{s}$) and spectral kurtosis ($Kurt_{s}$).

In this work, 12 (6 × 2) characteristics and 96 (6 × 2 × 8) characteristics are calculated, respectively, from the raw data and the EMD data. As the number of features is relatively high, a feature selection step is needed to choose only the best five features to use them as input for the classification methods. To carry on this process, a minimal subset of features that are required needs to be collected. This subset should be adequate to precisely differentiate between the healthy subjects and subjects with PD. Therefore, the use of a Random Forest feature selection method is key to obtaining the most relevant features from all the extracted features. In the Random Forest feature selection method, the score calculation phase includes the prediction performance of the model. It also reorders the features according to their calculated scores. The classifier inputs for the raw data and the EMD data are chosen as a set of five relevant features showing the best scores.

#### 4.1.1. Acquired Results Using Data Obtained

The acquired results are obtained using the features extracted from the raw data, the EMD data and both the EMD and the raw data together. In this example, a total of 48 (12 × 4), 384 (96 × 4) and 432 (12 × 4 + 96 × 4) features are used in the raw data, the EMD data and the raw and the EMD data, respectively.

Table 2 shows the results obtained using extracted features from the EMD data. What can be concluded from this table is that the SVM method results in the best performance when it comes to accuracy, precision, F-measure and recall. The RF approach and K-NN approach come after, by a slightly lower margin, whereas the CART approach shows the worst performance of all four methods. In this table we also see that the recognition rate is at least 78% and up to 91%.

Table 3 shows the results obtained using extracted features from the raw data. What can be concluded from this table is that the RF method results in the best performance when it comes to accuracy, precision, F-measure and recall. The SVM approach and the K-NN approach come after by a slightly lower margin, whereas the CART approach shows the worst performance of all four methods. In this table we also see that the recognition rate is at least 73% and up to 80%.

Table 4 shows the results obtained using extracted features from the raw data and the EMD data. What can be concluded from this table is that the RF method results in the best performance when it comes to accuracy, precision, F-measure and recall. The SVM approach and the K-NN approach come after, by a slightly lower margin, whereas the CART approach shows the worst performance of all four methods. In this table, we also see that the recognition rate is at least 80% and up to 94%.

**Figure 3:**
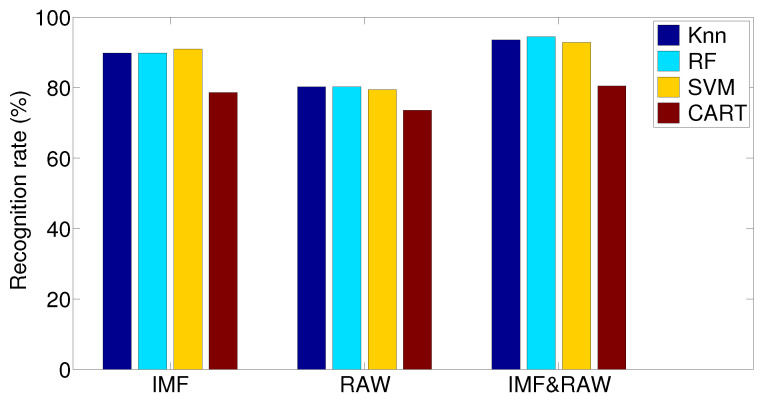
Obtained results in terms of recognition rate for each classifier using extracted/selected features from EMD, raw and EMD and raw data.

As a conclusion concerning the results obtained in the three tables and in Figure 3 focusing on the extracted features from the raw data, the EMD data and the raw and EMD data, the highest recognition rate is obtained using the EMD data or the EMD and the raw data. Features extracted only from the raw data gives the worst results in the given system.

#### 4.1.2. Obtained Results Using Classifier Combination Methods

As presented above, several classification methods were used to recognize healthy from PD subjects based on stabilometric data. These classifiers may give different decisions for the same observation. Therefore, combining the outputs (decisions) of these classifiers may lead to a significant improvement in the classification task. In the classifier combination context, the objective is not to reduce redundancy in the information from several classifiers (decisions), but instead, it is used in order to improve decision making. In this study, three well known methods of classifier combination are used and compared, namely: fusion based on Bayesian formalism method, fusion based on majority voting rule method and fusion based on Dempster–Sahfer formalism method.

Table 5 summarizes the results obtained using the classifier combination methods presented above. As shown in the table, the recognition rates obtained with the classifier combination methods are greater than 94%. These results show that the classifier combination methods allow improving the classification performance compared to those obtained with each classifier independently. By analyzing the combination results, it can be observed that the methods based on the Bayesian formalism and Dempster–Sahfer formalism present almost similar results and give better performance than the method based on majority vote. This can be explained by the fact that the Dempster–Sahfer and Bayes methods take into account the errors of each classifier, which is not the case for the majority vote method.

In order to analyze the confusion that can occur between classes, the global confusion matrix obtained using classifier combination based on Dempster–Sahfer formalism is given in Table 6. It can be observed that the healthy subjects are well classified with a correct classification rate of 97.32%. It can also be observed that in most cases the confusion is made between healthy and PD patients by considering the PD patients as healthy subjects with an error rate of 4.11%. This can be explained by the fact that some subjects are in the early stage of Parkinson’s disease and may be confused with healthy persons.

This study shows the performance of applying the EMD method on stabilometric data based on extracting temporal and spectral features from the resulting IMFs. The obtained results demonstrate the superiority of the proposed method (EMD-based method) over the classical method based on feature extraction directly from the raw data. In the proposed method, SVM succeeded in differentiating healthy from PD subjects in 91.08% of cases. It was capable of classifying correctly 91 out of 100 subjects. In addition, the developed strategy based on combining results from more than one classifier succeeded in improving the classification accuracy up to 96.51% for the Dempster–Sahfer method. This means that our approach provides a better classification of 95 out of 100 subjects. This result proves the superiority of the proposed method over the classical method that achieves in its best case an accuracy of 80.49%. Moreover, the 96.51% value should be acceptable from a clinical point of view because the margin error equal to 3.5% is very logical. According to that, this method can be used to help physicians diagnose PD disease and make decisions concerning an accurate and effective treatment.

### 4.2. Comparison with Other Studies

Mei et al. [58] present a taxonomy of the most relevant studies using machine-learning techniques for the Diagnosis of Parkinson’s Disease. They extracted 209 studies and investigated their aims, sources of data, types of data, machine-learning methods and associated outcomes.

The authors of [59] proposed a new approach for Parkinson’s disease classification based on data partitioning with the feature selection algorithm principal component analysis (PCA). They considered two classifications: healthy and Parkinson’s disease. They used a combination of SVM, and weighted k-NN classifiers and they obtained an accuracy of 89.23%. Celik et al. [60] proposed an approach to improve the diagnosis of Parkinson’s Disease using machine-learning methods. They performed a comparison between different classification methods such as Extra Trees, Logistic Regression, Gradient Boosting and Random Forest, and Support Vector Machine to predict Parkinson’s disease with 76% accuracy. Another study proposed by [61] consists of feature-selection and classification processes. For the feature-selection part, they used Feature Importance and Recursive Feature Elimination methods. For the classification process, they applied Classification and Regression Trees, Artificial Neural Networks and Support Vector Machines. They achieved an accuracy of 93.84%. Our proposed method provides an accuracy level of 96%.

## 5. Conclusions and Perspectives

In this paper, we introduced a strategy to classify healthy subjects and subjects with PD. The proposed approach consists of four main stages: stabilometric data decomposition using EMD, extraction of temporal and spectral features, selection of the features and classification using SVM, RF, KNN and CART methods. The obtained results show that the proposed approach can reach correct classification rates in up to 96% of cases in terms of classifying healthy and PD subjects. Using the EMD-based data, results show better classification rates than using classical strategies based on extracting features from raw data. This methodology could be used in future studies to distinguish between PD levels to help physicians detect the disease in earlier stages.

Two limitations can be mentioned: The first is the potential overfitting due to feature selection. The second is the comparability between the healthy subjects and the PD patients regarding age, and how close the PD population is to the target group of this model, mainly PD around the time of first diagnosis. As an extension of this study, the proposed methodology could be applied to a larger dataset for verification and validation purposes. A larger dataset should contain PD subjects from all age stages and from all PD levels to ensure that they represent the PD population. In addition, other feature selection algorithms should be applied to compare them and to choose the best one that gives the highest accuracy and avoid the over-fitting problems.

## Figures and Tables

**Table 1:** A description of the PD population.

Criteria	Value
Age (mean ± SD)	67 ± 8 years
Time since diagnosis	8 ± 5 years
Score (Hoenh and Yahr)	2.2 ± 0.3
Weight	75 ± 18 kg
Height	167 ± 11 cm

**Table 2:** $F_{1}$-measure per class, $F_{1}$-measure, precision, recall and average of accuracy rates (*R*) and its standard deviation (*std*) for each model (EMD).

	$F_{1}$ Measure	Precision	Recall	Accuracy (R)±(std)
k-NN (%)	89.90	90.31	90.19	89.90±4.51
CART (%)	78.51	78.98	78.20	78.71±3.81
RF (%)	90.01	90.12	0.16	90±3.16
SVM (%)	89.98	89.97	91.19	91.08±4.10

**Table 3:** $F_{1}$-measure per class, $F_{1}$-measure, precision, recall and average of accuracy rates (*R*) and its standard deviation (*std*) for each model (raw).

	$F_{1}$ Measure	Precision	Recall	Accuracy (R)±(std)
k-NN (%)	80.31	80.19	80.29	80.29±5.02
CART (%)	73.67	73.69	73.70	73.79±7.09
RF (%)	80.34	80.38	80.51	80.49±6.41
SVM (%)	79.60	80.21	79.39	79.79±5.61

**Table 4:** $F_{1}$-measure per class, $F_{1}$-measure, precision, recall and average of accuracy rates (*R*) and its standard deviation (*std*) for each model (EMD raw).

	$F_{1}$ Measure	Precision	Recall	Accuracy (R)±(std)
k-NN (%)	92.95	93.41	93.62	93.28±2.52
CART (%)	80.32	80.30	80.41	80.40±4.65
RF (%)	94.21	93.91	94.19	94.15±2.78
SVM (%)	92.51	92.48	92.8	92.6±2.13

**Table 5:** $F_{1}$-measure per class, $F_{1}$-measure, precision, recall and average of accuracy rates (*R*) and its standard deviation (*std*) for each model.

	$F_{1}$ Measure	Precision	Recall	Accuracy (R)±(std)
majority vote (%)	94.90	94.91	94.79	94.79±4.09
Bayes (%)	96.29	96.41	96.30	96.23±3.19
Dempster–Sahfer (%)	96.49	96.60	96.39	96.51±3.19

**Table 6:** Global confusion matrix obtained with Dempster–Sahfer classifier fusion.

		Healthy	PD Patients
True	Healthy (%)	97.32	2.68
classes	PD patients (%)	4.11	95.89

## Data Availability

Experimental Data was provided by the ARM Laboratory ARM, CHU Henri Mondor, Cŕeteil France.

## Abbreviations

The following abbreviations are used in this manuscript:

PD	Parkinson’s disease
EMD	Empirical mode decomposition
$IFM_{s}$	Intrinsic mode functions
KNN	K-Nearest Neighbor
SVM	Support Vector Machine
COP	Center of pressure
AR	Auto-regressive
AP	Anterior–posterior
ML	Medial–lateral
